# Induction of *ubiquitin C* (*UBC*) gene transcription is mediated by HSF1: role of proteotoxic and oxidative stress

**DOI:** 10.1002/2211-5463.12484

**Published:** 2018-07-24

**Authors:** Marzia Bianchi, Rita Crinelli, Vanessa Arbore, Mauro Magnani

**Affiliations:** ^1^ Department of Biomolecular Sciences, Biochemistry and Molecular Biology Section University of Urbino ‘Carlo Bo’ Italy

**Keywords:** HSF1, Nrf2, proteostasis, stress response, *UBC* gene, ubiquitin upregulation

## Abstract

The polyubiquitin gene *ubiquitin C* (*UBC*) is considered a stress protective gene and is upregulated under various stressful conditions, which is probably a consequence of an increased demand for ubiquitin in order to remove toxic misfolded proteins. We previously identified heat shock elements (HSEs) within the *UBC* promoter, which are responsible for heat shock factor (HSF)1‐driven induction of the *UBC* gene and are activated by proteotoxic stress. Here, we determined the molecular players driving the *UBC* gene transcriptional response to arsenite treatment, mainly addressing the role of the nuclear factor‐erythroid 2‐related factor 2 (Nrf2)‐mediated antioxidant pathway. Exposure of HeLa cells to arsenite caused a time‐dependent increase of *UBC* mRNA, while cell viability and proteasome activity were not affected. Nuclear accumulation of HSF1 and Nrf2 transcription factors was detected upon both arsenite and MG132 treatment, while HSF2 nuclear levels increased in MG132‐treated cells. Notably, siRNA‐mediated knockdown of Nrf2 did not reduce *UBC* transcription under either basal or stressful conditions, but significantly impaired the constitutive and inducible expression of well‐known antioxidant response element‐dependent genes. A chromatin immunoprecipitation assay consistently failed to detect Nrf2 binding to the *UBC* promoter sequence. By contrast, depletion of HSF1, but not HSF2, significantly compromised stress‐induced *UBC* expression. Critically, HSF1‐mediated *UBC trans*‐activation upon arsenite exposure relies on transcription factor binding to previously mapped distal HSEs, as demonstrated to occur under proteasome inhibition. These data highlight HSF1 as the pivotal transcription factor that translates different stress signals into *UBC* gene transcriptional induction.

**Abbreviations**
ARE: antioxidant response element
GCLC: glutamate‐cysteine ligase catalytic subunit
HMOX1: haem oxygenase‐1
HRP: horseradish peroxidase
HSE: heat shock element
HSF: heat shock factor
HSP: heat shock protein
HSR: heat shock response
Keap1: Kelch‐like ECH‐associated protein 1
LUC: luciferase
MEF: mouse embryonic fibroblast
MTS 
NaAsO_2_: sodium arsenite
NFE2L2: nuclear factor‐erythroid 2 like 2 (gene)
NQO1: NAD(P)H:quinone oxidoreductase‐1
Nrf2: nuclear factor‐erythroid 2‐related factor 2
ROS: reactive oxygen species
RT‐qPCR: real‐time quantitative polymerase chain reaction
Sp3: specificity protein 3
TFIID: transcription factor IID
TF: transcription factor
TSS: transcription start site
UBC: ubiquitin C or polyubiquitin‐C
Ub: ubiquitin
UPP: ubiquitin–proteasome pathway

Cells are constantly faced with different types of stress, such as heat shock, starvation, proteotoxic and oxidative stress, which can damage every cellular component, including nucleic acids and mainly proteins.

Under stress conditions proteins become damaged or unfolded and therefore need to be managed, because their accumulation is toxic to the cells. Proteome integrity is maintained by the so‐called proteostatic network, a multi‐compartmental highly interconnected system that assists proteins from synthesis to folding, trafficking and lastly degradation 1. In particular, under stress conditions, the molecular chaperones and the main degradation machinery, i.e. the ubiquitin–proteasome pathway (UPP), play a prominent role, respectively, in the repair and disposal of ‘non‐native’ proteins 2.

The UPP relies on the ubiquitin‐conjugating enzymes, the 26S proteasome, and of course on the signaling molecule ubiquitin (Ub). Ub is a highly conserved 76‐amino‐acid protein that exerts a myriad of diverse signaling functions, depending on the multiple ways by which it can be conjugated to other proteins (the ubiquitin code) 3, 4. The most widely understood outcome of ubiquitination is to tag intracellular proteins for proteasomal degradation, and this function is mostly accomplished by the Lys48‐linked polyUb chain signal 5, 6.

In the cell, Ub is dynamically distributed among distinct pools, which mainly include ‘free’ or unconjugated Ub, and ‘Ub–protein conjugates’, where the molecule Ub is peptide‐linked to its protein substrates 7, 8. The conjugate pool comprises both monoubiquitin conjugates and polyubiquitin conjugates; in addition, free polyubiquitin chains, which are not conjugated to substrates, also contribute to the total Ub cellular content 7. The distribution of Ub between the different pools is accomplished by Ub‐conjugating enzymes and deubiquitinating enzymes; the latter also carry out Ub recycling from the targeted substrates which are degraded by the proteasome 8. Although Ub has been so far considered an abundant protein inside the cells 9, different studies instead highlight how the Ub protein is not constitutively produced in excess; rather its levels are adjusted to meet ongoing cellular needs 10, 11, 12. This is exemplified by the increased Ub levels detected under stress conditions, when the cell needs to make more Ub to meet the tagging demand imposed by the abnormally high levels of misfolded proteins that have to be degraded by the proteasome 10, 13. Besides post‐translational mechanisms, levels of total cellular Ub are regulated by transcriptional control at the four different Ub coding loci, two of which encode Ub polyprotein (*UBB* and *UBC*) and the other two produce a fusion product of one Ub molecule and a ribosomal protein (*UbA52* and *UbS27A*) 14. We recently determined the contribution of the four Ub genes to the whole Ub transcriptome in cell cultures, before and after exposure to different stressors, including proteasome inhibition and oxidative stress: both *UBB* and *UBC* markedly contribute to maintaining Ub homeostasis under basal conditions, but they are at the front line in promptly providing the extra Ub needed in stressful conditions 15. Although *UBC* has long been known as a stress‐responsive gene, the molecular mechanisms driving the transcriptional induction provoked by stress exposure remained unexplored for a long time. Our research group has recently mapped and characterized the heat shock elements (HSEs) that orchestrate the transcriptional activation of the *UBC* gene under conditions of proteasome inhibition by MG132 16.

In the present study, the *trans*‐acting factors responsible for *UBC* gene transcriptional regulation were investigated in HeLa cells exposed to sodium arsenite (NaAsO_2_) in comparison with MG132 treatment. Indeed, Kim and coworkers reported that the upregulation of the *UBC* gene in mouse embryonic fibroblasts (MEFs) exposed to oxidative stress induced by arsenite occurred in a nuclear factor‐erythroid 2‐related factor 2 (Nrf2)‐dependent manner, and provided experimental evidence indicating that *UBC* is likely a direct target of Nrf2 17.

In fact, the main cellular defense against oxidative stress‐induced cytotoxicity relies on the activation of the Nrf2–Kelch‐like ECH‐associated protein 1 (Keap1) pathway 18. Under physiological conditions, Nrf2 protein levels are maintained low because of its rapid turnover thanks to the interaction with the ubiquitin E3 ligase adaptor Keap1, which promotes Nrf2 ubiquitination and proteasomal degradation 18. Keap1, due to critical Cys residues, acts as a redox sensor so that under oxidative stress it becomes inactive and fails to present Nrf2 to the UPP: as a result, newly synthesized Nrf2 translocates to the nucleus, interacts with small Maf proteins and together they bind to antioxidant response elements (AREs), eliciting the transcriptional activation of target genes 19, 20.

Besides its well‐ascertained role as the master regulator of the antioxidant cell response, Nrf2 has more recently emerged as a key component of the transduction machinery to maintain proteostasis, by acting both as a sensor for the emergency signals derived from misfolded protein accumulation and as an effector able to upregulate the expression of several proteasome‐ and ubiquitination‐related genes 21.

The polyubiquitin genes *UBB* and *UBC* are themselves among the upregulated genes under oxidative stress conditions 13, 15, 17, although the need of ubiquitination to degrade oxidized proteins is still controversial, with the majority of publications suggesting that they are degraded by the 20S proteasome independent of Ub 2, 22. Thus, in light of these findings and open questions, we asked whether and to what extent Nrf2 mediates *UBC* upregulation under stress conditions, possibly cooperating with heat‐shock factors (HSFs) previously reported to be essential for *UBC* transcriptional induction. Our results provide evidence that the stress‐inducible polyubiquitin gene *UBC* is likely not a direct target of the Nrf2‐mediated antioxidant pathway, while being consistent with and strongly supporting a prominent role of HSF1 in driving *UBC* upregulation under different stress conditions.

## Materials and methods

### Cell culture and treatments

Human cervical adenocarcinoma HeLa and murine NIH3T3 cell lines were obtained from ATCC. HeLa cells were grown in RPMI 1640 supplemented with 10% heat‐inactivated fetal bovine serum (Thermo Fisher Scientific, Waltham, MA, USA), 2 mm glutamine and 1× antibiotics (100 μg·mL^−1^ streptomycin and 100 U·mL^−1^ penicillin) at 5% CO_2_ at 37 °C. NIH3T3 cells were grown in Dulbecco's modified Eagle's medium (DMEM) supplemented with 10% heat‐inactivated fetal bovine serum (Thermo Fisher Scientific), 2 mm glutamine, 1× non‐essential amino acids and 1× antibiotics, at 5% CO_2_ and 37 °C. Upon reaching around 90% confluence, cells were harvested using trypsin–EDTA and diluted at a subcultivation ratio of ~ 1 : 6. For gene expression studies, HeLa cells were plated in six‐well plates at a density of 3 × 10^5^ cells/well and treated with different concentrations (from 10 to 80 μm) of sodium arsenite (NaAsO_2_) or with 20 μm proteasome inhibitor MG132 (Selleckchem, Munich, Germany) for up to 8 h; untreated cells or cells treated with the vehicle DMSO at 0.04% (v/v) were used, respectively, as control. All the chemicals and cell culture supplements were purchased from Sigma‐Aldrich (St. Louis, MO, USA), unless otherwise specified. For chromatin immunoprecipitation experiments, cells plated in 60 mm Petri dishes, at a density of 10^6^ cells/dish, were challenged with stressors (80 μm NaAsO_2_ or 20 μm MG132) for 4 h or left not treated, for the control sample.

### Small interfering RNA transfection in HeLa and NIH3T3 cells

Small interfering RNA (siRNA)‐mediated gene silencing in HeLa and NIH3T3 cells was achieved by transfecting siRNA duplexes with the HighPerfect reagent (Qiagen Inc., Valencia, CA, USA), using either the standard or the fast‐forward transfection protocol, according to the manufacturer's guidelines. For HeLa cells, we trypsinized cells from an actively growing culture and immediately transfected 2.2 × 10^5^ cells (fast‐forward protocol); alternatively 1.5 × 10^5^ cells were seeded in each well of a six‐well plate the day before transfection (standard protocol). siRNA targeting HSF1, HSF2 or Nrf2 mRNA and the GFP‐targeting control siRNA were transfected at a final concentration of 10 nm in the presence of 12 μL of HighPerfect transfection reagent, for each well. Transfected cells were incubated 48 h before 8 h NaAsO_2_ or MG132 treatment. HSF1, HSF2 and Nrf2 siRNAs were purchased from Qiagen, while the GFP control siRNA was from Biomers (Ulm, Germany).

The oligonucleotide targeting sequences are reported below:

Hs_HSF1: CAGGTTGTTCATAGTCAGAAT

Hs_HSF2: AAGACGTTTATTCATGTTCAA and CTGCGCCGCGTTAACAATGAA

Hs_NFE2L2 (nuclear factor‐erythroid 2 Like 2): CCCATTGATGTTTCTGATCTA

GFP: CGGCAAGCTGACCCTGAAGTTCAT

As for NIH3T3, 3.2 × 10^5^ cells were transfected with Nrf2 siRNA (Sigma‐Aldrich) or GFP targeting control siRNA at 5 nm final concentration, following the fast‐forward protocol.

The oligonucleotide targeting sequence for mouse Nrf2 was as reported in 17:

Mm_NFE2L2: CCAAAGCTAGTATAGCAATAA

Cells were incubated 48 h post‐transfection before 8 h treatment with 80 μm NaAsO_2_ or 20 μm MG132; transfected unstressed cells served as control.

### Real‐time quantitative polymerase chain reaction

For gene‐specific expression analysis, total RNA was isolated using the RNeasy Plus Mini kit (Qiagen). Five hundred nanograms of total RNA was reverse transcribed using PrimeScript™ RT Master Mix (Perfect Real Time; Takara Bio Europe SAS, Saint‐Germain‐en‐Laye, France) with oligo‐dT and random 6‐mer primers, following the manufacturer's instructions. qPCR detection and expression analysis of genes was performed using the SYBR green quantitative real‐time PCR assay, with the Hot‐Rescue Real Time PCR Kit (Diatheva s.r.l., Cartoceto PU, Italy), essentially according to the manufacturer's instructions. Briefly, the reaction was set up in a 25 μL final volume, using 5 ng cDNA as template and 200 nm of each specific primer. For real‐time quantitative polymerase chain reaction (RT‐qPCR) amplifications, we ran 40 PCR cycles using the following thermal profile: 15 s 95 °C melting temperature, 15 s 60 °C annealing and 1 min 72 °C extension temperature per cycle; before cycling, 10 min 95 °C was allowed for Hot‐Rescue Taq DNA polymerase activation. Fluorescence intensity of each amplified sample was measured with an ABI PRISM 7700 Sequence detection system (Applied Biosystems, Foster City, CA, USA). All measurements were performed at least in triplicate and reported as the average values ± standard error of the mean (mean ± SEM). Target gene values were normalized with glyceraldehyde 3‐phosphate dehydrogenase (GAPDH) mRNA measurements and expression data were calculated according to the 2^−ΔΔ*C*^ ^t^ method 23. Primers were designed using primer3 plus 24 and their sequences are reported in Table 1.

**Table 1:** Primers designed for real‐time qPCR expression assays. FWD, forward; m, murine; REV, reverse

Gene target	FWD primer sequence (5′ → 3′)	REV primer sequence (5′ → 3′)
*GAPDH*	TGCACCACCAACTGCTTAG	GATGCAGGGATGATGTTC
*UBC*	GTGTCTAAGTTTCCCCTTTTAAGG	TTGGGAATGCAACAACTTTATTG
*NFE2L2*	AAACCAGTGGATCTGCCAAC	ACGTAGCCGAAGAAACCTCA
*HSF1*	CTGACGGACGTGCAGCTGAT	CCCGCCACAGAGCCTCAT
*HSF2*	CCAGAATGGCCAAAGTTTTCTG	GCTTGCCATATTATTGTGCTTGAA
*GCLC*	CTGTTGCAGGAAGGCATTGAT	TTCAAACAGTGTCAGTGGGTCTCT
*HMOX1*	CCAGCAACAAAGTGCAAGATTC	TCACATGGCATAAAGCCCTACAG
*HSP70*	AGCTGAAGAAGGGTCAAGTGAC	TGGATAGGGCAAATCCTGAG
*LUC*	TGTACACGTTCGTCACATCTCATCT	AGTGCAATTGTCTTGTCCCTATCG
*mGAPDH*	GGCATTGCTCTCAATGACAA	CTTGCTCAGTGTCCTTGCTG
*mNFE2L2*	CCAGACAGACACCAGTGGATC	GGCAGTGAAGACTGAACTTTCAG
*mUBC*	CCAGTGTTACCACCAAGAAGGT	AATGCAAGAACTTTATTCAAAGTGC
*mGCLC*	AGGCTCTCTGCACCATCACT	CTCTGGGTTGGGTCTGTGTT
*mHMOX1*	TGCTCGAATGAACACTCTGG	TCCTCTGTCAGCATCACCTG

### Reporter construct transient transfection

The wild‐type (P1) and mutant reporter constructs (P1 mut FR1‐2 and P1 mut FR6) used in this study have been previously described 16. They contain a *UBC* promoter fragment spanning from −916 (upstream of the transcription start site (TSS)) to +876 (including first exon and the unique intron). The distal or proximal HSF binding motifs mapped in the upstream sequence have been mutagenized to obtain P1 mut FR1‐2 and P1 mut FR6, respectively. HeLa cells seeded in six‐well plates (3 × 10^5^ cells/well) the day before transfection were transiently transfected with 400 ng of DNA/well by using Effectene transfection reagent (Qiagen), according to the manufacturer's protocol. Forty‐eight hours after transfection, cells were treated with 80 μm NaAsO_2_, 20 μm MG132 or neither for 8 h, and then luciferase expression was detected by real‐time qPCR. Luciferase mRNA was normalized to GAPDH mRNA and the fold induction compared with the untreated wild‐type reporter plasmid.

### Cell extracts

After treatment, cells were washed in PBS and lysed by sonication in sodium dodecyl sulfate (SDS) buffer containing 50 mm Tris/HCl pH 8.0, 2% (w/v) SDS, 10 mm *N*‐ethylmaleimide supplemented with a cocktail of protease inhibitors (Roche Diagnostics, Mannheim, Germany). Lysates were boiled and then cleared by centrifugation at 12 000 ***g***. The protein content was determined according to Lowry, using bovine serum albumin as standard. Nuclear extracts were obtained by low salt/detergent cell lysis followed by high salt extraction of nuclei as previously described with some modifications 25. Briefly, after washing with PBS, cells were scraped from the dishes with cold buffer A [10 mm HEPES/KOH, pH 7.9, 10 mm KCl and 0.1% Nonidet‐P40, supplemented with protease and phosphatase inhibitors (1 mm NaF, 1 mm Na_3_VO_4_)]. The cell suspension was then chilled on ice for 10 min before centrifugation at 14 000 ***g***. The resultant nuclear pellet was resuspended in cold buffer B (20 mm HEPES/KOH, pH 7.9, 25% glycerol, 0.42 m NaCl containing protease and phosphatase inhibitors) and incubated on ice for 20 min before being centrifuged at 14 000 ***g***. Nuclear proteins were collected in the supernatant, diluted 1 : 3 in buffer C (20 mm HEPES/KOH, pH 7.9, 20% glycerol, 50 mm KCl containing protease and phosphatase inhibitors) and protein concentration was determined by the Bradford assay.

### Western blot analysis

Proteins were resolved by SDS/PAGE and immunodetected with the following primary antibodies: anti‐HSF1 (4356, Cell Signaling Technology, Danvers, MA, USA), anti‐HSF2 (H300) (sc‐13056, Santa Cruz Biotechnology, Dallas, TX, USA), anti‐Nrf2 (D1Z9C) (12721, Cell Signaling Technology). Anti‐TFIID (TBP, 58C9 sc‐421 Santa Cruz Biotechnology) and/or anti‐specificity protein 3 (Sp3) (sc‐644, Santa Cruz Biotechnology) were used as nuclear loading control, while anti‐actin (A 2066, Sigma‐Aldrich) was used to demonstrate equal protein loading in whole cell extracts. Briefly, gels were electroblotted onto a nitrocellulose membrane (0.2 mm pore size) (Bio‐Rad, Hercules, CA, USA). The blots were probed with the primary antibodies listed above and bands were detected by horseradish peroxidase (HRP)‐conjugated secondary antibody (Bio‐Rad). Peroxidase activity was detected with the enhanced chemiluminescence detection method (WesternBright ECL, Advasta, Menlo Park, CA, USA).

### Chromatin immunoprecipitation and PCR of chromatin templates

To perform ChIP, we used the ChIP Assay kit (Upstate Biotechnology Inc., New York, NY, USA), essentially according to the manufacturer's instructions, as described in 26. Briefly, HeLa cells were treated with 80 μm NaAsO_2_, 20 μm MG132 for 4 h at 37 °C or left untreated (control). Nuclear proteins were cross‐linked to DNA by adding 1% formaldehyde (final concentration) directly to the medium. The cross‐linking was stopped by 0.125 m glycine. Chromatin of cross‐linked cells was fragmented to an average size of 200–500 bp with a Labsonic 1510 Sonicator (Braun, Melsungen, Germany) by performing 10–12 pulses, 15 s on, 45 s off, at 45 watts. Sheared chromatin, corresponding to 2 × 10^6^ cell equivalents, was immunoprecipitated, overnight at 4 °C, with 10 μg of anti‐Nrf2 (C‐20) X (sc‐722X, Santa Cruz Biotechnology) or with 10 μg of non‐specific IgG (anti‐rabbit IgG, Upstate Biotechnology), as control. Immunoprecipitated DNA (ChIPed DNA) and the input DNA (1% of chromatin withdrawn before the immunoprecipitation step) were extracted with the spin columns provided by the kit and then used as a template for real‐time quantitative PCR using promoter region‐specific primers of *UBC* (FR1‐FWD 5′‐GAGAAATTTCCATGCCTCCCTGTT‐3′ and FR1‐RE V 5′‐AAAAGAGGCGGAAACCCCACA‐3′; FR5‐FWD 5′‐GCTGCCACGTCAGACGA‐3′ and FR5‐REV 5′‐ AAGGCCGAGTCTTATGAGCA‐3′; FR6‐FWD 5′‐CTCGGCCTTAGAACCCCAGTATC‐3′ and FR6‐REV 5′‐AACTAGCTGTGCCACACCCG‐3′) and *NQO1* (FWD 5′‐TCCAAATCCGCAGTCACAG‐3′ and REV 5′‐CTTGGCACGAAATGGAGC‐3′), and Hot‐Rescue Real Time PCR Kit (Diatheva s.r.l.).

Cycling conditions were as described above for gene expression studies. Data were analyzed using the formula 2^−Δ*C*^ ^t^ × 100, where Δ*C*_t_ = *C*_t,output_−*C*_t,input_ (output means the ChIPed DNA).

### Cell viability assay

The effect of stress treatments on cell viability was evaluated by seeding 1.5 × 10^4^ HeLa cells/well in 96‐well plates in complete RPMI medium. The day after, fresh medium containing the appropriate NaAsO_2_ and MG132 concentration was added and incubation at 37 °C extended up to 8 h. Cell viability was assessed by using the CellTiter 96^®^ AQueous One Solution Cell Proliferation Assay (Promega s.r.l., Milano, Italy). This assay is based on the reduction of the MTS reagent [3‐(4,5‐dimethylthiazol‐2yl)‐5‐(3‐carboxymethoxyphenyl)‐2‐(4‐sulfophenyl)2*H*‐tetrazolium, inner salt] into a colored formazan product that is soluble in tissue culture medium. This conversion is accomplished by NADPH or NADH produced by dehydrogenase enzymes in metabolically active cells. The quantity of formazan product, as measured by the absorbance at 490 nm, is directly proportional to the number of living cells in culture.

### Proteasome activity assay

The chymotrypsin‐like activity of the 20S proteasome was measured in cell lysates using the fluorogenic substrate *N*‐succinyl‐Leu‐Leu‐Val‐Tyr‐7‐amido‐4‐methylcoumarin (sLLVY‐NH‐Mec, Sigma‐Aldrich) as previously described 27. Briefly, cells treated 4 and 8 h with the stressors (NaAsO_2_ and MG132) and untreated HeLa cells (control) were homogenized on ice in a buffer consisting of 50 mm HEPES/KOH pH 7.8, 1 mm dithiothreitol and 0.25 m sucrose. Twenty micrograms of cleared extracts were incubated at 37 °C in 100 mm HEPES/KOH buffer, pH 7.8, 5 mm MgCl_2_ and 10 mm KCl and the reaction was initiated by addition of 0.2 mm fluorogenic substrate. The breakdown of the peptide was monitored using a fluorescence microplate reader (FLUOstar OPTIMA, BMG Labtech GmbH, Offenburg, Germany) with an excitation wavelength of 355 nm and an emission wavelength of 460 nm. Proteasome activity in each sample, expressed as fluorimetric units·min^−1^·mg^−1^, was calculated by submitting data to linear regression analysis (*R* ^2^ > 0.99).

### Statistical analysis

Statistical analyses were performed with prism software (GraphPad Software, La Jolla, CA, USA). Statistical significance was evaluated by the two‐tail paired Student's *t* test for pairwise comparisons or one‐way ANOVA with Tukey's *post hoc* test for multiple comparisons. Results are expressed as means ± SEM and differences are considered significant for *P* < 0.05.

## Results

### *UBC* gene induction by both NaAsO_2_ and MG132 is paralleled by HSFs and Nrf2 nuclear translocation

**Figure 1:**
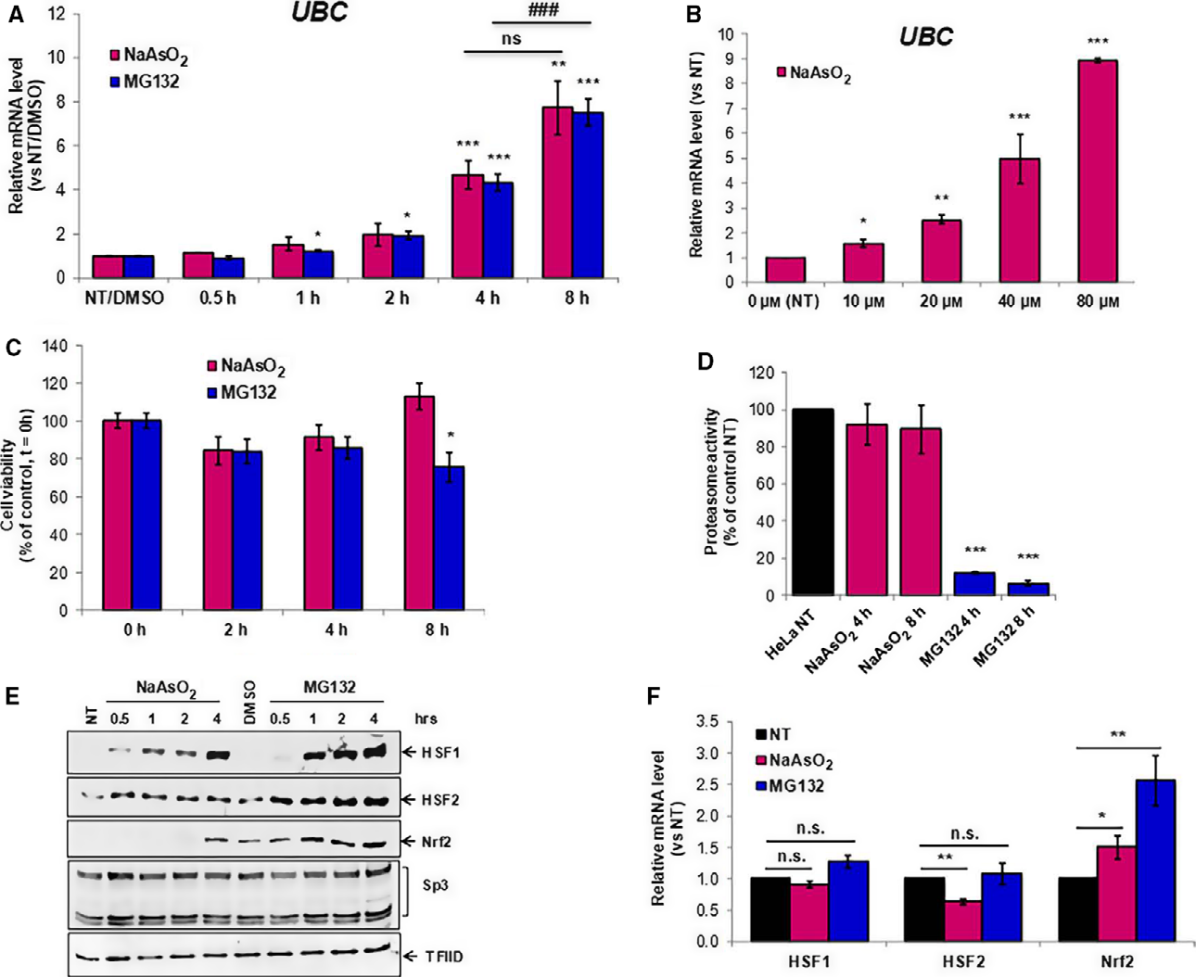
Arsenite and proteasome inhibitor MG132 induce *UBC* gene expression and promote nuclear accumulation of HSF1, HSF2 and Nrf2 transcription factors. (A) HeLa cells were treated with NaAsO_2_ (80 μm) or MG132 (20 μm) over an 8‐h time course (as indicated). *UBC* mRNA levels were determined by RT‐qPCR, normalized to *GAPDH* levels and expressed as fold increase relative to control cells (NT/DMSO). (B) HeLa cells treated for 8 h with different concentrations of NaAsO_2_ (as indicated) were analyzed as in (A). *UBC* mRNA level is shown as fold increase *versus* the untreated sample (NT). (C) Cell viability of HeLa cells exposed to NaAsO_2_ or MG132 for the indicated times (*n* = 3 each) were evaluated by the MTS assay and shown as a percentage with respect to the untreated‐cell value (time 0 h), set equal to 100. (D) Cells treated with NaAsO_2_ or MG132 for 4 and 8 h and untreated cells (NT) were subjected to the proteasome activity assay (*n* = 4). (E) Nuclear levels of HSF1, HSF2 and Nrf2 transcription factors upon cell treatment with stressors for the indicated times were analyzed by western immunoblotting of nuclear fractions, with anti‐HSF1, anti‐HSF2 and anti‐Nrf2 specific antibodies. TFIID and Sp3 were used as nuclear loading controls. Arrows indicate the detected protein. (F) HSF1, HSF2 and Nrf2 mRNA levels in cells treated for 8 h with NaAsO_2_ or MG132 (*n* = 3 each) were measured by RT‐qPCR and expressed as a fold change relative to NT cells. All data are expressed as means ± SEM of the indicated number of samples. Asterisks denote statistical significance, calculated by one‐way ANOVA,* versus* control as specified or indicated by bars; *P < 0.05; **P < 0.01; ***, ###*P* < 0.001; n.s., not significant.

HeLa cells were exposed to 80 μm NaAsO_2_ or 20 μm of the proteasome inhibitor MG132. We monitored the *UBC* gene response in the presence of both stressors by quantitative real‐time PCR at different time points (Fig. 1A). Both proteasome inhibitor and arsenite treatment led to a gradual upregulation of the *UBC* gene over the 8 h incubation period, in line with previous results 15. However, the trend was slightly different from a statistical point of view. MG132‐treated cells revealed a statistically significant increase of *UBC* transcript, *versus* dimethyl sulfoxide (DMSO)‐treated cells, after just 1 h (1.3‐fold increase) up to 8 h (7.5‐fold induction). Sodium arsenite caused a significant increase in *UBC* transcription after 4 h of treatment (4.7‐fold induction with respect to untreated HeLa cells) and although the highest induction was observed at 8 h (a 7.8‐fold increase), this was not statistically significant with respect to the 4 h value. The *UBC* gene response to different NaAsO_2_ concentrations, from 10 to 80 μm, was analyzed: a significant increase in the *UBC* transcript level was already detected upon cell treatment with 10 μm NaAsO_2_, and moreover the upregulation showed a clear dose‐dependent response (Fig. 1B). It is worth noting that the concentration of NaAsO_2_ was then chosen in order to maximize the stress‐induced *UBC* transcription, without affecting cell viability. An MTS assay performed on HeLa cells challenged with the stressors *versus* control cells indeed revealed that arsenite exposure did not affect cell viability over the 8 h period, while MG132 treatment resulted in a slightly reduced cell viability with respect to the vehicle DMSO‐treated cells, at the longer time point (Fig. 1C). While MG132 is expected to block proteasome activity and induce proteotoxic stress 16, arsenite is considered a typical inducer of oxidative stress that in turn promotes protein damage 28, 29. In addition, arsenic has been demonstrated to bind to sulfhydryl groups causing protein dysfunction 30. Finally, arsenite treatment has been reported to damage the proteasome and reduce its activity 17. Therefore, to assess whether under our experimental conditions arsenite could induce *UBC* upregulation through a mechanism similar to MG132, i.e. by blocking the proteasome, the 20S chymotrypsin‐like activity was assayed in cell extracts derived from control or arsenite‐treated HeLa cells. Proteasome activity was not significantly affected by NaAsO_2_ treatment, whereas it was blunted by the inhibitor MG132 (Fig. 1D).

In an attempt to search for the transcription factor(s) (TFs) behind the *UBC* gene upregulation upon arsenite‐triggered oxidative stress and proteasome inhibition, we focused our investigation on the HSFs that are at the front line to face proteotoxic stress by eliciting the so‐called ‘heat shock response’ (HSR) and on Nrf2, which is considered the master regulator of the oxidative stress response.

Therefore, we sought to determine whether *UBC* induction was accompanied by an accumulation of HSFs, in particular HSF1 and HSF2, and Nrf2 proteins in the nucleus of cells exposed to NaAsO_2_ or MG132. HeLa cells were treated with both stressors in a time course experiment and appropriate control samples were also established; the nuclear extracts prepared from stressed and control cells, were subjected to immunoblot analysis with specific antibodies. Of note, the nuclear extracts did not exhibit immunostaining for IκBα, a mostly cytosolic protein (data not shown), but stained positive for the nuclear transcription factors TFIID and Sp3 (Fig. 1E), demonstrating the efficacy of the fractionation method. As shown in Fig. 1E, HSF1 was undetectable in the nuclei of untreated or DMSO‐receiving cells, while it appeared in the nuclei within 0.5 h of treatment with either NaAsO_2_ or MG132 and its levels increased up to the 4 h time point with both stressors. On the contrary, HSF2 was already present in the nuclear compartment and its levels were unaffected by arsenite treatment, but increased in MG132‐treated cells consistent with its mechanism of activation that involves protein stabilization. In the MG132‐treated cells, Nrf2 nuclear accumulation started within 1 h of treatment with the proteasome inhibitor, and the maximal level was attained at 4 h. Although nuclear accumulation of Nrf2 was observed in the DMSO‐treated sample with respect to the untreated control, Nrf2 induction was significantly increased by MG132 exposure as compared with the DMSO control. In arsenite‐treated cells, the nuclear translocation of Nrf2 was instead detectable only at the longer time point (4 h).

To determine whether the nuclear accumulation of HSFs and Nrf2 observed upon stress was only due to post‐transcriptional mechanisms or was also the result of an increase in gene transcription, HSF1, HSF2 and Nrf2 mRNA levels in HeLa cells exposed for 8 h to NaAsO_2_ or MG132 were evaluated. HSF1 mRNA levels were found to be unaffected by the two stressors, HSF2 mRNA decreased in arsenite‐treated cells, while the Nrf2 mRNA displayed a statistically significant increase after both sodium arsenite and MG132 treatment (1.5‐ and 2.6‐fold increase, respectively) (Fig. 1F).

**Figure 2:**
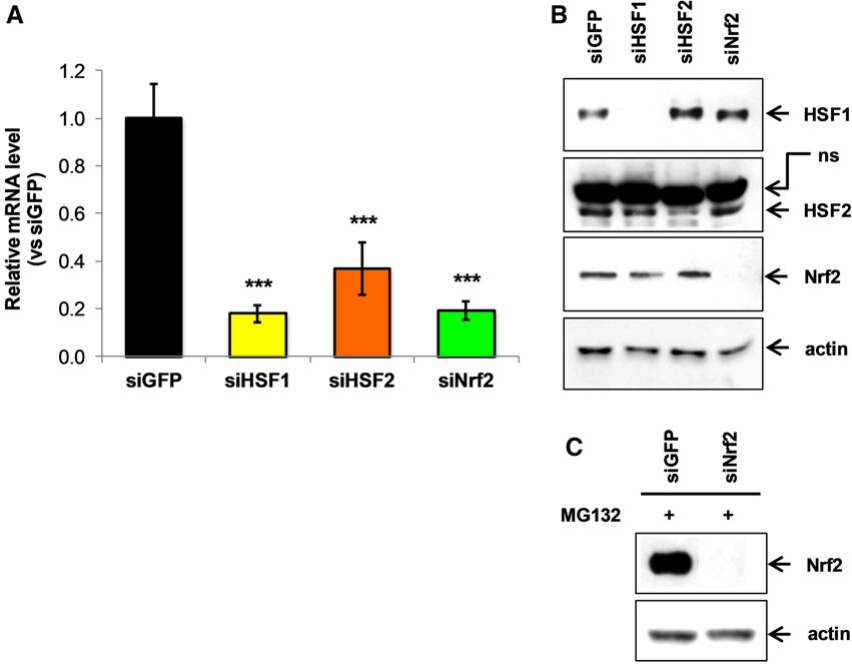
siRNA‐mediated knockdown of HSF1, HSF2 and Nrf2 transcription factors. (A) HeLa cells were transiently transfected with control (GFP), HSF1, HSF2 or Nrf2 siRNAs (indicated as siGFP, siHSF1, siHSF2, siNrf2). At 48 h after transfection the mRNA levels of knockdown genes were measured by quantitative real‐time PCR. The results are normalized to *GAPDH* mRNA and depicted as fold change compared with siGFP transfected cells. Data are means ± SEM (*n* = 11). Asterisks denote statistical significance, calculated by Student's *t* test, *versus* GFP control siRNA; ***P < 0.001. (B) Western immunoblot of total proteins from HeLa cells transfected with the indicated siRNA, at 48 h post‐transfection. Equal amounts of total cellular proteins were loaded and probed with antibodies specific for HSF1, HSF2 and Nrf2. Blot was reprobed with anti‐actin as a loading control. (C) Nrf2 expression (relative to actin) in cells transfected with control GFP siRNA or siRNA targeting Nrf2, after 8 h treatment with 20 μm MG132. Arrows indicate the detected protein; ns, not specific.

To define the role of HSF1 and ‐2 and Nrf2 in *UBC* transcriptional upregulation upon NaAsO_2_‐ or MG132‐triggered stressful conditions, and their possible crosstalk, we depleted each protein factor by siRNA‐mediated knockdown and then analyzed the *UBC* gene response to stress. After transient silencing, HeLa cells showed 82% and 81% reduction of HSF1 and Nrf2 transcripts compared with cells transfected with control siRNA directed against GFP mRNA, while a lower downmodulation was achieved for HSF2, using two different targeting oligonucleotides (63% *versus* GFP control siRNA) (Fig. 2A). The efficacy and specificity of the silencing strategy were also evaluated at the protein level, by western immunoblotting on whole cellular extracts: HSF1 and Nrf2 protein bands were both undetectable upon specific siRNA transfection, with no evidence of off‐target effects (Fig. 2B). The silencing approach was still efficient under stress conditions leading to Nrf2 protein stabilization: in fact Nrf2‐silenced HeLa cells did not exhibit increased Nrf2 protein levels upon exposure to MG132 (Fig. 2C). Regarding HSF2, a faint residual anti‐HSF2 immunoreactive band was observed in siHSF2 transfected cells (Fig. 2B), according to RT‐qPCR data.

### Nrf2 is not required for the stress‐induced *UBC* gene expression in human HeLa cells and murine NIH3T3 cells

**Figure 3:**
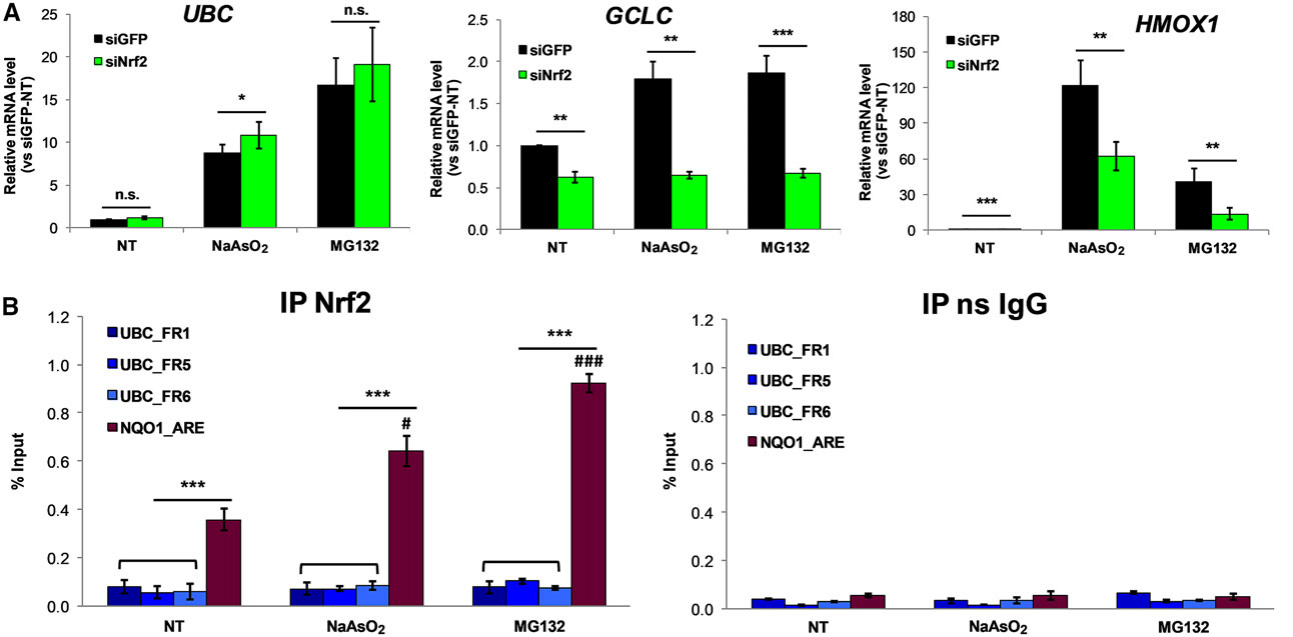
*UBC* gene induction by oxidative and proteotoxic stress is independent of Nrf2. (A) HeLa cells receiving control GFP siRNA (siGFP) or Nrf2 specific siRNA (siNrf2) were treated, 48 h after transfection, with 80 μm NaAsO_2_ or 20 μm MG132 for 8 h or left untreated as control (NT). *UBC*,*GCLC* and *HMOX1* mRNAs were measured by RT‐qPCR, normalized to *GAPDH* levels and expressed as fold increase relative to untreated siGFP‐transfected cells (*n* = 6). (B) ChIP‐PCR analysis of HeLa cells before (NT) or after 4 h exposure to NaAsO_2_ or MG132 (same concentration as in (A)). Chromatin was immunoprecipitated with Nrf2 specific antibody or with non‐specific anti‐rabbit IgG (as internal IP control). RT‐qPCR was performed on the immunoprecipitated samples (IP Nrf2 and IP ns IgG) and on the chromatin before immunoprecipitation (Input), using primers that amplify three different *UBC* promoter regions (FR1 −916/−759; FR5 −278/−187; FR6 −196/−96). Amplification with primers specific for an ARE‐containing locus in the *NQO1* promoter served as positive control. Binding is depicted as the percentage of input values and is the mean of three independent experiments. Data are means ± SEM of the indicated experiments. Statistical analysis was performed by Student's *t* test (A) or one‐way ANOVA (B). Asterisks denote statistical significance (as indicated by bars) *P < 0.05; **P < 0.01; ***P < 0.001; n.s., not significant. #*P* < 0.05 and ###*P* < 0.001 indicate statistical significance *versus NQO1*_ARE NT sample.

Depletion of Nrf2 did not affect the stress‐induced *UBC* gene expression in human HeLa cells exposed to either NaAsO_2_ or MG132; rather the *UBC* transcriptional response was further increased (Fig. 3A). However, Nrf2 knockdown led to a significantly reduced expression of well‐known Nrf2 target genes, such as glutamate‐cysteine ligase catalytic subunit (*GCLC*) and haem oxygenase‐1 (*HMOX1*) (Fig. 3A), thus confirming the efficacy of the silencing strategy. Moreover, for both genes we observed mRNA reduction with Nrf2 silencing at both baseline and after stress treatments, in accordance with the demonstrated ability of Nrf2 to control constitutive and inducible expression of many ARE‐driven genes 19, 20, 31. *In silico* analyses of the *UBC* promoter sequence, by using different bioinformatics tools, was performed to search for putative ARE‐binding sites. matinspector 32, tess 33 and tfsearch (http://diyhpl.us/~bryan/irc/protocol-online/protocol-cache/TFSEARCH.html) did not find AREs in the *UBC* promoter sequence, while pscan 34 detected a putative Nrf2 binding site approximately 200 nt upstream to the TSS. To gain insight into the relevance of this finding in intact cells, we used chromatin immunoprecipitation (ChIP) to check for Nrf2 binding to the 1 kb promoter sequence upstream of the TSS of *UBC*, in NaAsO_2_‐ and MG132‐treated cells. Detection of Nrf2 binding at the known ARE‐binding loci in the promoter region of a prototypic Nrf2‐dependent enzyme, NAD(P)H:quinone oxidoreductase‐1 (*NQO1*), was used as a positive control for Nrf2 activation. Indeed, Nrf2 occupancy at the *NQO1* promoter was observed already in untreated cells, as demonstrated by the percentage input being consistently higher with respect to the corresponding non‐specific IgG control; after stress treatment Nrf2 binding further increased, with a higher enrichment observed in the MG132‐treated sample (Fig. 3B). However, no significant enrichment with respect to the non‐specific IgG sample was detected when the *UBC* promoter region was amplified by three different primer pairs encompassing the sequence spanning from −916 to −96 upstream to the TSS, in both basal conditions and after stress treatment (Fig. 3B). As a whole the ChIP data indicate that Nrf2 does not bind to the 1 kb *UBC* promoter region screened and therefore is unlikely to participate to the *UBC* gene transcriptional upregulation upon stress challenge, in close agreement with the output of the Nrf2 silencing strategy.

**Figure 4:**
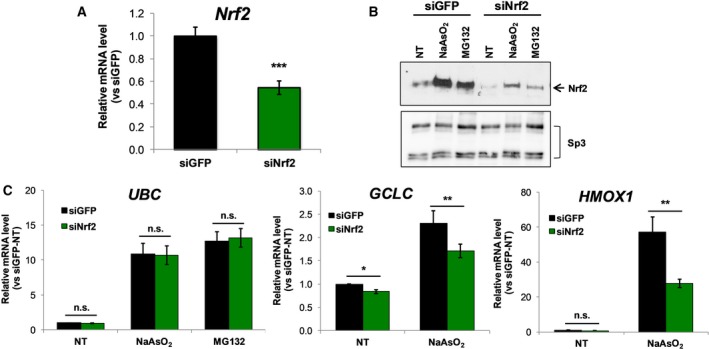
Downmodulation of Nrf2 does not impair *UBC* gene induction by oxidative and proteotoxic stress in the mouse cell line NIH3T3. (A) NIH3T3 cells were transiently transfected with Nrf2 targeting siRNA and control GFP siRNA. At 48 h after transfection Nrf2 mRNA levels were measured by RT‐qPCR, normalized to GAPDH mRNA and depicted as fold change *versus* GFP siRNA transfected cells (*n* = 5). (B) siNrf2 and siGFP transfected cells were subjected to 8 h treatment with 80 μm NaAsO_2_ or 20 μm MG132 or left untreated (NT) and Nrf2 protein levels were determined by immunoblot analysis of nuclear extracts with anti‐Nrf2 specific antibody. Sp3 was stained as nuclear loading control. Arrows indicate the detected protein. (C) *UBC* mRNA as well as *GCLC* and *HMOX1* mRNAs were measured by RT‐qPCR, in NIH3T3 cells treated as indicated. The mRNA levels of analyzed genes were normalized to GAPDH levels and expressed as fold change relative to untreated siGFP transfected cells (*n* = 5). Data shown in (A,C) are the means ± SEM from the indicated number of samples. Asterisks denote statistical significance, calculated by Student's *t* test, *versus* control as indicated by bars; *P < 0.05; **P < 0.01; ***P < 0.001; n.s., not significant.

Since Kim and coworkers 17 provided evidence that *UBC* may be a direct target of Nrf2 in mouse embryonic fibroblasts exposed to arsenite, we next sought to investigate the role of Nrf2 in *UBC* induction in NIH3T3, another standardized mouse embryonic fibroblast cell line 35. Nrf2 knockdown was obtained by transient siRNA transfection; the expression of Nrf2 mRNA was reduced by only 46% with respect to GFP siRNA receiving cells (Fig. 4A); however, the level of Nrf2 protein in the nuclei was drastically reduced upon specific Nrf2 silencing (Fig. 4B, upper panel). Moreover, the Nrf2 targeting siRNA was able to counteract the transcription factor accumulation upon NaAsO_2_ and MG132 treatment, thus confirming the efficacy of the siRNA strategy (Fig. 4B). However, as for the human HeLa cells, transfection of Nrf2 siRNA into the murine NIH3T3 cells had no impact on the *UBC* mRNA induction elicited by the two stressors investigated and also basal *UBC* expression was not significantly affected (Fig. 4C), whereas siNrf2 transfection significantly reduced the arsenite‐inducible expression of two prototypic Nrf2‐regulated genes, *GCLC* and *HMOX1*, by 26% and 52%, respectively (Fig. 4C). *GCLC*, but not *HMOX1*, basal expression was likewise reduced by Nrf2 knockdown (Fig. 4C). Taken as a whole, these data suggest that Nrf2 does not seem to play a role in the transcriptional regulation of polyubiquitin gene *UBC*, at least in the murine cell line NIH3T3, exposed to arsenite as stress inducer.

### Arsenite‐induced *UBC* gene expression is HSF1‐dependent

**Figure 5:**
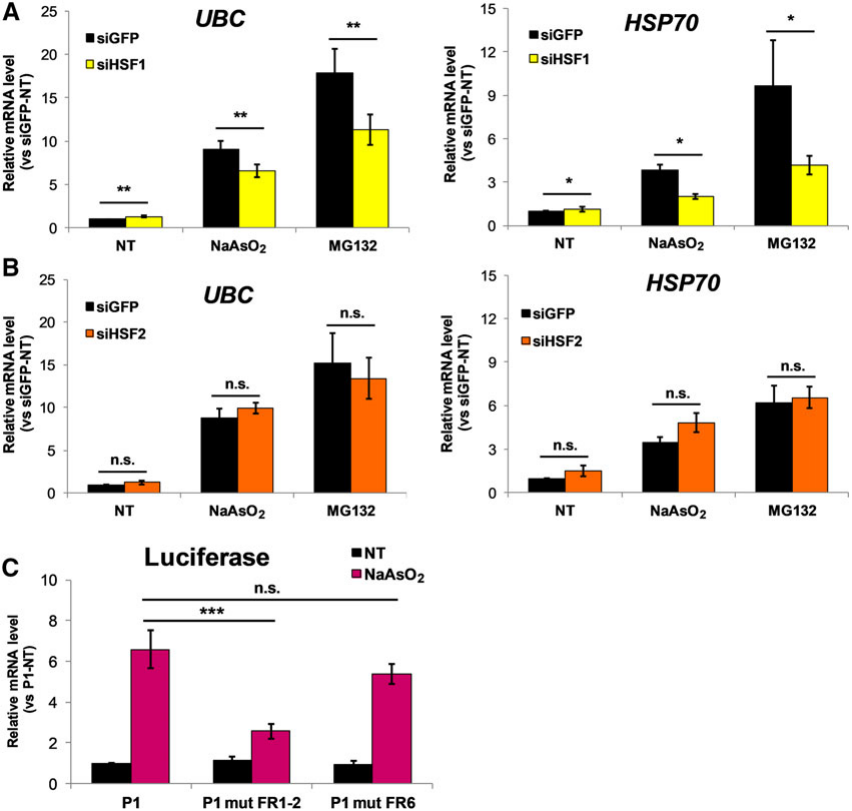
*UBC* gene induction by oxidative and proteotoxic stress is dependent on HSF1. (A,B) HeLa cells were transfected with either HSF1 and HSF2 specific siRNAs or control GFP siRNA. Forty‐eight hours after transfection, cells were treated with 80 μm NaAsO_2_ or 20 μm MG132 for 8 h or left not treated as control (NT). *UBC* and *HSP70* mRNAs were measured by RT‐qPCR, normalized to *GAPDH* levels and expressed as fold increase relative to not treated siGFP‐transfected cells. Data are means ± SEM of six experiments; asterisks denote statistical significance, calculated by Student's *t* test (as indicated by bars): *P < 0.05; **P < 0.01; n.s., not significant. (C) Expression analysis of promoter luciferase‐reporter constructs was performed in HeLa cells transiently transfected with the wild‐type construct P1 and its mutant derivatives (P1 mut FR1‐2 and P1 mut FR6) and treated, 48 h post‐transfection, with 80 μm NaAsO_2_ for 8 h or with no stressor added. Luciferase mRNA was detected by RT‐qPCR and expression data, normalized to GAPDH, were compared with the value obtained for the untreated wild‐type construct (P1, NT) (*n* = 6). Bars indicate significant differences between P1 and P1 mutant‐driven luciferase expression, upon arsenite exposure, calculated by one‐way ANOVA. ***P < 0.001; n.s., not significant.

Having excluded a direct role for Nrf2, the involvement of HSF1 and HSF2 in the stress‐induced upregulation of *UBC* was further investigated. To this end, we used the siRNA approach as for Nrf2. Transfection with siHSF1, but not siHSF2, significantly compromised the inducible expression of *UBC* mRNA by NaAsO_2_ and MG132 (respectively, 28% and 37% reduction in siHSF1‐transfected cells compared with siGFP‐transfected cells) (Fig. 5A,B). Basal *UBC* expression was positively affected by HSF1 siRNA (Fig. 5A), while transfection with HSF2 siRNA had no impact (Fig. 5B). Constitutive and inducible expression of heat shock protein (HSP)70 was measured as a positive control. The HSP70 transcript level displayed a similar layout to that of *UBC* mRNA, both at basal and in stressful conditions, with both HSF‐targeting siRNAs (Fig. 5A,B). Overall, these data point towards a prominent role of HSF1 in mediating *UBC* gene response to oxidative stress induced by arsenite. To get deeper insight into the role of HSF1, we tested the responsiveness to NaAsO_2_ of three reporter constructs previously generated, where luciferase (LUC) expression is driven by a *UBC* promoter fragment containing three HSEs, two distal (FR1, FR2) and one proximal (FR6) to the TSS, that we have mapped and functionally characterized 16. Luciferase expression driven by the wild‐type promoter sequence (P1) showed a 6.6‐fold increase upon arsenite exposure; mutations of the distal HSF binding motifs (P1 mut FR1‐2) significantly impaired the arsenite‐induced upregulation of reporter gene transcription (2.6‐fold increase *versus* untreated sample) (Fig. 5C). Of note, mutations in the proximal HSE (P1 mut FR6) did not affect the inducible activity of the promoter construct (5.4‐fold increase in luciferase mRNA level, which is not statistically different from the one detected for the wild‐type construct P1) (Fig. 5C).

## Discussion

The bulk of the knowledge accumulated on the Ub molecule's structure and functions, which led to the deciphering of the so‐called ‘ubiquitin code’, and the wide characterization of the ‘ubiquitin‐related proteome’ have not been accompanied by a concomitant increase in knowledge of the molecular mechanisms regulating Ub gene expression under basal and stressful conditions. With respect to the regulation of polyubiquitin gene *UBC*, some pieces of information have been recently added by our own studies, highlighting the role of the YY1 transcription factor and the importance of the presence of the intron in driving the basal promoter activity 26. Moreover, in agreement with data published by Vihervaara *et al*. 36 for ChIPseq analyses revealing HSF1 and HSF2 binding to the *UBC* promoter upon heat stress, we mapped and characterized the HSEs lying in the *UBC* promoter sequence, demonstrating how these sequences affect *UBC* upregulation, in response to cell treatment with the proteasome inhibitor MG132, by possibly binding HSF1 and HSF2 16.

In the present study, the role of HSFs and Nrf2 in the *UBC* gene induction upon cell treatment with different stressors (the proteasome inhibitor MG132 and sodium arsenite) was thoroughly investigated.

Nrf2 is considered the master regulator of the antioxidant cell response 18. In the canonical pathway of activation, Nrf2 must detach from its redox‐sensor inhibitor, Keap1, to shuttle to the nucleus and activate transcription of ARE‐driven genes 37, 38. However, recent studies have begun to unravel a broader impact of this transcription factor on the modulation of proteostasis, by controlling genes related to the unfolded protein response, autophagy and notably the proteasomal pathways 21, 39.

Here we found that Nrf2 accumulates in the nuclei of HeLa cells treated with arsenite and also in those treated with MG132. Since Nrf2 is a short‐lived protein and its degradation is dependent on the UPP, when the proteasomal activity is almost totally inhibited, the transcription factor becomes stabilized and shuttles to the nucleus 40, 41. Intriguingly, MG132 and the proteasome inhibitor bortezomib, employed clinically, have been reported to induce oxidative stress, which provokes the accumulation of unfolded/damaged proteins 42, 43. Although the molecular mechanisms underlying reactive oxygen species (ROS) generation after proteasome inhibition are still unclear, this stressor can mimic the classical Nrf2 inducers. Likewise arsenite, besides induction of ROS 28, 29, has been demonstrated to cause proteotoxic stress, by directly binding to proteins 30. It is noteworthy that the proteasome catalytic activity is not affected by the arsenite treatment in HeLa cells suggesting that, under our experimental conditions, Nrf2 escapes Keap1‐mediated degradation 21, 38.

However, Nrf2 knockdown did not impair the stress‐induced *UBC* gene expression in HeLa cells. Bioinformatic analysis of the *UBC* promoter sequence with different tools (matinspector 32, tess 33 and tfsearch (http://diyhpl.us/~bryan/irc/protocol-online/protocol-cache/TFSEARCH.html)) did not reveal Nrf2 binding sites, with the only exception of pscan 34, which detected a putative ARE in the *UBC* promoter sequence, approximately 200 nt upstream to the TSS. Since the main drawback of these methods lies in the high number of predictions that prove to be ‘false positive’, we were aware that the presumption of transcription factor occupancy needed to be confirmed by more direct experimental evidence, such as ChIP.

In support of the pscan output, a genome‐wide ChIP‐Seq experiment in lymphoid cells treated with sulforaphane found a high‐confidence Nrf2‐binding site near (−271) the TSS of the *UBC* gene [44; https://www.ncbi.nlm.nih.gov/geo/query/acc.cgi?acc=GSE37589].

Therefore, to evaluate possible Nrf2 binding, *in vivo*, to the predicted ARE in the proximal *UBC* promoter, we performed the more definitive ChIP assay, in HeLa cells challenged with stressors (NaAsO_2_ and MG132) *versus* untreated ones (as a control). ChIP‐PCR analysis using three different primer pairs that fall in the −916 to −96 *UBC* promoter sequence proved that Nrf2 does not bind to this *UBC* promoter region either in basal conditions or after stress exposure. These results are consistent with those obtained by siRNA‐mediated Nrf2 knockdown. Of note, the putative Nrf2 binding site predicted by pscan software falls in the amplified FR5 region, for which a percentage input similar to the one obtained for the non‐specific IgG control was detected. As a whole, the aforementioned results indicate that Nrf2 is dispensable for the *UBC* gene upregulation elicited by the two stressors.

Kim and coworkers investigated the role of the Nrf2‐dependent pathway in the upregulation of polyubiquitin gene *UBC* in mouse embryonic fibroblasts, under arsenite treatment 17. The authors concluded that the *UBC* gene, in mice, is likely a direct target of Nrf2 17, supporting their evidences with results of global mapping of Nrf2‐binding sites obtained by a ChIP‐Seq experiment, performed on the same cell model 45.

In an attempt to reconcile the published data that argue for an Nrf2‐dependent *UBC* upregulation under oxidative stress in MEFs 17 and our evidence reported herein for the human *UBC* gene, which seems to overshadow Nrf2 participation, we used the mouse fibroblast cell line NIH3T3 to investigate the *UBC* gene response to arsenite‐ and MG132‐triggered stress, and the involvement of Nrf2. As for human HeLa cells, Nrf2 silencing in NIH3T3 did not compromise either *UBC* gene basal expression or its transcriptional induction upon stress treatments. This may be explained by the different ‘not‐primary’ cell line used (NIH3T3 *versus* MEFs) and/or by the less effective silencing of Nrf2 in NIH3T3, although the reduction of Nrf2 protein levels in the nuclei of siNrf2‐transfected cells was sufficient to significantly impair the stress‐induced transcription of the well‐characterized ARE‐driven genes *GCLC* and *HMOX1*.

siRNA‐mediated knockdown of HSF1 effectively suppressed *UBC* upregulation by the arsenite and MG132‐treated cells, proving that HSF1 is required for the *UBC* gene response to stress, as reported by Vihervaara *et al*. 36. On the contrary, HSF2 silencing did not affect the *UBC* gene stress response, despite the TF being stabilized and accumulating in the nucleus upon proteasome inhibition; furthermore, it was previously found to bind to the HSEs in the *UBC* promoter, under MG132‐triggered proteotoxic stress 16. Although the shared consensus DNA binding sequences might suggest an intimate interplay between HSF1 and HSF2 in transcriptional regulation, they are indeed characterized by different regulatory mechanisms (mainly post‐translational modifications for HSF1 and control at the expression level for HSF2, 46) and have been reported to play different roles in the transcriptional response to stress, with HSF1 emerging as a potent transactivator that drives a rapid stress response 36.

Transfection of luciferase reporter constructs herein revealed that HSF1‐dependent *UBC* transcriptional induction upon arsenite exposure relies on TF binding to the distal HSEs, previously mapped on the *UBC* promoter region, spanning up to 1 kb upstream to the TSS 16. This means that HSF1 orchestrates *UBC* gene transcriptional induction upon both MG132 and arsenite stressors, by binding to the same regulatory target motifs. Of course, this does not exclude possible HSF1 binding to other more distal *cis*‐elements, to regulate the *UBC* gene expression under stressful conditions. The HSF1‐dependent upregulation of heat‐shock genes in response to environmental redox changes, caused by typical Nrf2/Keap1 inducers, has been documented 47. Different reports indeed describe the shared role of Nrf2 and HSF1 in affecting the cellular redox state by promoting the reduced state; this means, for example, that the electrophilic compounds, namely molecules able to directly or indirectly react with sulfhydryl groups, are among the stressors that can activate the heat shock response 47, 48, 49. However, while for Nrf2 the oxidative sensor has been well characterized and attributed to the interactor protein Keap1, bearing critical cysteine residues that are the target of the oxidant molecules, for HSF1 this task can be performed by its main negative regulators, the molecular chaperones HSP70 and HSP90 48. Moreover, it has been speculated that in some cases the activators could be sensed by the transcription factor itself; in fact the oxidation of specific cysteine residues engaged in redox‐sensitive disulfide bonds within HSF1 has been reported to negatively affect its DNA binding activity 50, 51. However, the identity of the primary cellular sensor of sulfhydryl reactive activators, namely the Keap1 counterpart, that triggers HSF1 release from its inhibitors in mammalian cells has yet to be established.

The results reported herein highlight that, whatever the underlying mechanism, in HeLa cells, arsenite‐induced stress upregulates the *UBC* gene in an HSF1‐dependent, but Nrf2‐independent, manner. This transcriptional response provides the extra Ub needed to manage the cell stress response, even though the need of Ub to degrade oxidized proteins is still debated 2, 22, 52.

HSF1 is a key player of the transcriptional programs mounted in stressed cells to maintain proteostasis, being the prime coordinator of the HSR, which is accomplished by HSF1‐mediated expression of stress‐protective genes. However, despite its name, the heat shock transcription factor 1 is activated not only in response to elevated temperatures, but also upon cell exposure to oxidants, metals and other conditions that cause protein misfolding 53, 54.

On the other side, *UBC* is by far known as a heat shock responsive gene and, as such, it is an HSF1 target gene; indeed, its transcriptional upregulation under proteotoxic conditions (induced by heat stress or proteasome inhibition) is primarily sustained by HSF1 binding to the HSEs identified in the gene promoter 16, 36.

Arsenite‐induced stress at the molecular level shares many features with the heat shock response and, as such, arsenite has been reported to induce heat shock proteins to save the proteome from stress injury, which requires a functional HSF1 55. Intriguingly, oxidative stress, which is typical of arsenic‐related effects, has been reported to directly affect different components of the UPP 2. Moreover, cellular ROS generation does not explain all the arsenic‐linked effects; in fact, direct protein binding by arsenicals can result in protein dysfunction 30. In this complex scenario, the identification of the molecular players that drive *UBC* gene upregulation under arsenite‐triggered cellular stress is not trivial and cannot be predicted on the bases of studies performed with other cellular stressors.

The present paper uncovers that HSF1‐mediated *trans*‐activation is required for *UBC* induction in cells challenged with arsenite, which was not previously known. This makes HSF1 the master modulator of *UBC* gene responsiveness to diverse stresses. Although the molecular mechanism(s) by which HSF1 senses the different stressors, becomes activated and conveys the detected signals to the responsive gene promoters remain to be elucidated, our findings represent a new breakthrough to better understand how the *UBC* gene is upregulated to strengthen the cellular responses to induced stress. The mechanisms envisaged may also foster translational research, facilitating the development of new therapeutics to modulate both HSF1 function and *UBC* gene expression.

## Author contributions

MB and RC conceived and designed the project. MB, RC and VA acquired the data. MB, RC, VA and MM analyzed and interpreted the data. MB wrote the paper.
